# Supplementary material for: Unveiling the Bioactive Potential of the Invasive Jellyfish Phyllorhiza punctata Through Integrative Transcriptomic and Proteomic Analyses
Source: Biomolecules. 2025 Aug 4;15(8):1121. doi: 10.3390/biom15081121 (PMC12383608; doi:10.3390/biom15081121)
Supplement: Supplementary file 1 [file biomolecules-15-01121-s001.zip › Suplementary Material/FileS3/PEPFOLD4/XP_065056582_10656/clusters.html]

 PEP-FOLD clusters


Download archive of all models  here   

|  |  |  |
| --- | --- | --- |
| # | file | sOPEP |
| # cluster 1 - Size: 1 |  |  |
|  | XP\_065056582\_10656-model1 | -18.7836 |
| # cluster 2 - Size: 1 |  |  |
|  | XP\_065056582\_10656-model2 | -5.8153 |
| # cluster 3 - Size: 1 |  |  |
|  | XP\_065056582\_10656-model3 | -5.63641 |
| # cluster 4 - Size: 1 |  |  |
|  | XP\_065056582\_10656-model4 | -5.47631 |
| # cluster 5 - Size: 1 |  |  |
|  | XP\_065056582\_10656-model5 | -5.43739 |
| # cluster 6 - Size: 1 |  |  |
|  | XP\_065056582\_10656-model6 | -5.34205 |
| # cluster 7 - Size: 4 |  |  |
|  | XP\_065056582\_10656-model7 | -5.25013 |
|  | XP\_065056582\_10656-model7.1 | -4.98655 |
|  | XP\_065056582\_10656-model7.2 | -4.94287 |
|  | XP\_065056582\_10656-model7.3 | -4.70486 |
| # cluster 8 - Size: 2 |  |  |
|  | XP\_065056582\_10656-model8 | -5.23698 |
|  | XP\_065056582\_10656-model8.1 | -5.04776 |
| # cluster 9 - Size: 1 |  |  |
|  | XP\_065056582\_10656-model9 | -5.22289 |
| # cluster 10 - Size: 2 |  |  |
|  | XP\_065056582\_10656-model10 | -5.2134 |
|  | XP\_065056582\_10656-model10.1 | -5.09588 |
| # cluster 11 - Size: 1 |  |  |
|  | XP\_065056582\_10656-model11 | -5.17257 |
| # cluster 12 - Size: 1 |  |  |
|  | XP\_065056582\_10656-model12 | -5.12723 |
| # cluster 13 - Size: 1 |  |  |
|  | XP\_065056582\_10656-model13 | -5.08637 |
| # cluster 14 - Size: 1 |  |  |
|  | XP\_065056582\_10656-model14 | -5.08067 |
| # cluster 15 - Size: 3 |  |  |
|  | XP\_065056582\_10656-model15 | -5.06891 |
|  | XP\_065056582\_10656-model15.1 | -4.99344 |
|  | XP\_065056582\_10656-model15.2 | -4.78344 |
| # cluster 16 - Size: 8 |  |  |
|  | XP\_065056582\_10656-model16 | -5.06154 |
|  | XP\_065056582\_10656-model16.1 | -4.92114 |
|  | XP\_065056582\_10656-model16.2 | -4.85506 |
|  | XP\_065056582\_10656-model16.3 | -4.81233 |
|  | XP\_065056582\_10656-model16.4 | -4.78399 |
|  | XP\_065056582\_10656-model16.5 | -4.73769 |
|  | XP\_065056582\_10656-model16.6 | -4.7287 |
|  | XP\_065056582\_10656-model16.7 | -4.67685 |
| # cluster 17 - Size: 2 |  |  |
|  | XP\_065056582\_10656-model17 | -5.03429 |
|  | XP\_065056582\_10656-model17.1 | -4.83578 |
| # cluster 18 - Size: 1 |  |  |
|  | XP\_065056582\_10656-model18 | -5.0196 |
| # cluster 19 - Size: 1 |  |  |
|  | XP\_065056582\_10656-model19 | -5.00373 |
| # cluster 20 - Size: 3 |  |  |
|  | XP\_065056582\_10656-model20 | -5.00205 |
|  | XP\_065056582\_10656-model20.1 | -4.89014 |
|  | XP\_065056582\_10656-model20.2 | -4.85049 |
| # cluster 21 - Size: 3 |  |  |
|  | XP\_065056582\_10656-model21 | -4.99042 |
|  | XP\_065056582\_10656-model21.1 | -4.91602 |
|  | XP\_065056582\_10656-model21.2 | -4.8247 |
| # cluster 22 - Size: 1 |  |  |
|  | XP\_065056582\_10656-model22 | -4.98232 |
| # cluster 23 - Size: 2 |  |  |
|  | XP\_065056582\_10656-model23 | -4.97207 |
|  | XP\_065056582\_10656-model23.1 | -4.96018 |
| # cluster 24 - Size: 4 |  |  |
|  | XP\_065056582\_10656-model24 | -4.96983 |
|  | XP\_065056582\_10656-model24.1 | -4.92785 |
|  | XP\_065056582\_10656-model24.2 | -4.87481 |
|  | XP\_065056582\_10656-model24.3 | -4.77369 |
| # cluster 25 - Size: 3 |  |  |
|  | XP\_065056582\_10656-model25 | -4.94662 |
|  | XP\_065056582\_10656-model25.1 | -4.83815 |
|  | XP\_065056582\_10656-model25.2 | -4.81828 |
| # cluster 26 - Size: 1 |  |  |
|  | XP\_065056582\_10656-model26 | -4.91969 |
| # cluster 27 - Size: 11 |  |  |
|  | XP\_065056582\_10656-model27 | -4.91459 |
|  | XP\_065056582\_10656-model27.1 | -4.9012 |
|  | XP\_065056582\_10656-model27.2 | -4.88598 |
|  | XP\_065056582\_10656-model27.3 | -4.83339 |
|  | XP\_065056582\_10656-model27.4 | -4.80683 |
|  | XP\_065056582\_10656-model27.5 | -4.78583 |
|  | XP\_065056582\_10656-model27.6 | -4.77692 |
|  | XP\_065056582\_10656-model27.7 | -4.77316 |
|  | XP\_065056582\_10656-model27.8 | -4.7302 |
|  | XP\_065056582\_10656-model27.9 | -4.68494 |
|  | XP\_065056582\_10656-model27.10 | -4.63257 |
| # cluster 28 - Size: 5 |  |  |
|  | XP\_065056582\_10656-model28 | -4.91456 |
|  | XP\_065056582\_10656-model28.1 | -4.89333 |
|  | XP\_065056582\_10656-model28.2 | -4.71188 |
|  | XP\_065056582\_10656-model28.3 | -4.67899 |
|  | XP\_065056582\_10656-model28.4 | -4.64717 |
| # cluster 29 - Size: 3 |  |  |
|  | XP\_065056582\_10656-model29 | -4.89883 |
|  | XP\_065056582\_10656-model29.1 | -4.85115 |
|  | XP\_065056582\_10656-model29.2 | -4.7248 |
| # cluster 30 - Size: 1 |  |  |
|  | XP\_065056582\_10656-model30 | -4.8972 |
| # cluster 31 - Size: 2 |  |  |
|  | XP\_065056582\_10656-model31 | -4.87787 |
|  | XP\_065056582\_10656-model31.1 | -4.76644 |
| # cluster 32 - Size: 4 |  |  |
|  | XP\_065056582\_10656-model32 | -4.87139 |
|  | XP\_065056582\_10656-model32.1 | -4.81011 |
|  | XP\_065056582\_10656-model32.2 | -4.77886 |
|  | XP\_065056582\_10656-model32.3 | -4.749 |
| # cluster 33 - Size: 1 |  |  |
|  | XP\_065056582\_10656-model33 | -4.86388 |
| # cluster 34 - Size: 1 |  |  |
|  | XP\_065056582\_10656-model34 | -4.86054 |
| # cluster 35 - Size: 2 |  |  |
|  | XP\_065056582\_10656-model35 | -4.85568 |
|  | XP\_065056582\_10656-model35.1 | -4.82937 |
| # cluster 36 - Size: 2 |  |  |
|  | XP\_065056582\_10656-model36 | -4.84852 |
|  | XP\_065056582\_10656-model36.1 | -4.76422 |
| # cluster 37 - Size: 2 |  |  |
|  | XP\_065056582\_10656-model37 | -4.83722 |
|  | XP\_065056582\_10656-model37.1 | -4.686 |
| # cluster 38 - Size: 3 |  |  |
|  | XP\_065056582\_10656-model38 | -4.83706 |
|  | XP\_065056582\_10656-model38.1 | -4.83471 |
|  | XP\_065056582\_10656-model38.2 | -4.81827 |
| # cluster 39 - Size: 2 |  |  |
|  | XP\_065056582\_10656-model39 | -4.82441 |
|  | XP\_065056582\_10656-model39.1 | -4.67033 |
| # cluster 40 - Size: 2 |  |  |
|  | XP\_065056582\_10656-model40 | -4.82043 |
|  | XP\_065056582\_10656-model40.1 | -4.78786 |
| # cluster 41 - Size: 1 |  |  |
|  | XP\_065056582\_10656-model41 | -4.81358 |
| # cluster 42 - Size: 1 |  |  |
|  | XP\_065056582\_10656-model42 | -4.80505 |
| # cluster 43 - Size: 2 |  |  |
|  | XP\_065056582\_10656-model43 | -4.80414 |
|  | XP\_065056582\_10656-model43.1 | -4.71918 |
| # cluster 44 - Size: 1 |  |  |
|  | XP\_065056582\_10656-model44 | -4.79679 |
| # cluster 45 - Size: 1 |  |  |
|  | XP\_065056582\_10656-model45 | -4.78703 |
| # cluster 46 - Size: 1 |  |  |
|  | XP\_065056582\_10656-model46 | -4.7531 |
| # cluster 47 - Size: 1 |  |  |
|  | XP\_065056582\_10656-model47 | -4.67848 |
|  |  |  |
